# Guidelines adherence in the treatment of patients with newly diagnosed type 2 diabetes: a historical cohort comparing the use of metformin in Quebec pre and post-Canadian Diabetes Association guidelines

**DOI:** 10.1186/1472-6963-13-442

**Published:** 2013-10-25

**Authors:** Ting-Yu Wang, Tewodros Eguale, Robyn Tamblyn

**Affiliations:** 1 Department of Medicine, McGill University, Montreal, Quebec, Canada; 2 Department of Endocrinology, McGill University, Montreal, Quebec, Canada; 3 Department of Epidemiology, Biostatistics and Occupational Health, McGill University, Montreal, Quebec, Canada

**Keywords:** Type 2 diabetes, Metformin, Guidelines adherence, Medical informatics

## Abstract

**Background:**

Given the high prevalence of diabetes, guidelines are updated frequently to reflect optimal treatment recommendations. Our study aims to measure the response of primary care physicians to changes in choice of initial therapy for patients with type 2 diabetes in relationship to a change in Canadian Diabetes Association (CDA) Guidelines in 2008. We also assessed patients’ and physicians’ factors which may affect this change.

**Methods:**

Historical cohort study of primary care physicians’ participating in an electronic medical record research network in Quebec, Canada. 111 primary care physicians and 1279 newly treated patients with diabetes with a prescription of an oral hypoglycemic agent (OHA) between January 20 2003 and December 29 2011 were included. Multivariate GEE logistic regression was used to estimate the impact of guideline change on treatment choice controlling for patients’ and physicians’ characteristics.

**Results:**

After the new CDA guidelines, there was an increase in incident use of metformin from 89.7% to 94.6% (OR 1.86, 95% CI 1.20-2.90) with an accompanying reduction in the use of thiazolidinediones (OR 0.21, 95% CI 0.08-0.55), and reduction in the initiation of sulfonylureas (OR 0.78, 95% CI 0.43-1.09). Physicians’ attitudes to evidence-based practice did not significantly modify response to a change in guidelines recommendations. However, older patients and those with renal failure were less likely to receive metformin.

**Conclusions:**

Metformin initiation in newly diagnosed diabetes patients has increased post 2008 CDA guidelines. However, due to the nature of the study design, we can not determine whether the observed change in metformin prescribing was causally related to the change in the guideline.

## Background

Over the last few decades, demographic and lifestyle changes have together contributed to the growing burden of diabetes worldwide. In Canada alone, more than 9 million individuals have been diagnosed with diabetes or pre-diabetes and over 40,000 Canadians die annually with diabetes as a contributing factor [1]. The disease is associated with excess morbidities — including atherosclerosis, nephropathy, retinopathy, cardiovascular disease and depression [2,3] — as well as early mortality, therefore leading to high socio-economic cost. Recent estimates suggest that by 2020, diabetes will cost nearly 16.9 billion (CAD) annually [1]. Given the complexity of chronic diseases, guidelines are developed to synthesize new scientific evidence into recommendations that will facilitate optimal disease management. However in many instances, patients do not achieve recommended care [4-6]. In diabetes, guidelines are published regularly but published studies have revealed low adherence to guidelines (either due to patients’ non-compliance or physicians’ non-adherence) [7-9].

In its latest clinical practice guidelines (October 2008) [10], the Canadian Diabetes Association (CDA) recommended metformin as the initiating treatment for all patients newly diagnosed with type 2 diabetes, regardless of individual characteristics. The change was made based on its effectiveness in lowering blood glucose, its relatively mild side effect profile (no hypoglycemia or weight gain) and its demonstrated benefit in overweight patients [11]. This differed from the previous CDA guidelines (2003), which had recommended starting with a sulfonylurea, an alpha-glucosidase inhibitor, or metformin for individuals with a BMI of less than 25, and metformin specifically for individuals with a BMI of over 25 [12]. Prior to the most recent (2008) guidelines, a population-based administrative database study of seniors showed that approximately 75% of diabetics aged 65 or over in Ontario were prescribed metformin as their first oral hypoglycemic agent (OHA) [13]. Due to the simplified 2008 CDA guidelines, we would expect to see an increase in its initiation rate. We used this opportunity of a guideline change to estimate the change in physicians’ approach to the treatment of diabetes in response to guidelines changes as well as patients’ and physicians’ factors that may influence guidelines adherence.

## Methods

### Context

The study was conducted in Quebec, Canada, where all residents have insurance for medical and hospital care through the Régie de l’Assurance Maladie du Québec (RAMQ). Prescription drugs are covered by the provincial insurance agency for seniors (age over 65) [14], social assistance recipients, and those not insured through their employer. In 2003, MOXXI, an experimental community-based electronic health record and clinical information system, was the first to link to these databases and integrate this information into electronic health record systems to support clinical decision-making [15]. The MOXXI electronic health record includes: 1) a problem list that is automatically generated from diagnostic codes within daily updates of medical services claims, treatment indications entered at the time of drug prescribing, and manual entries; 2) a drug profile that shows prescribed and dispensed drugs, ED visits and hospitalizations in the past 12 months, and 3) an electronic prescribing tool that requires mandatory entry of treatment indication (from a list of on- and off-label indications), and documentation of the reason for drug discontinuations and dose changes. Data generated by the MOXXI electronic health record have been validated and used in a number of studies [15-18]. The MOXXI system provided us with the first opportunity to investigate how changes in CDA guideline recommendation for initial treatment of type 2 diabetes affected physician prescribing practices. This study has been approved by our institutional review board (McGill University Institutional Review Board).

### Design and study population

A dynamic historical cohort study was conducted comparing newly treated patients with type 2 diabetes in the pre-2008 guidelines period with those started on therapy in the post-2008 period by family physicians in the MOXXI system. Among the patients participating in the MOXXI research program, patients were eligible if they had a diagnosis of type 2 diabetes, and were newly started on an OHA between 2003 and 2011, defined as having no prior use of an antidiabetic agent in the past two years. Patients’ diagnosis of type 2 diabetes was defined based on their prescription of an OHA and a treatment indication for diabetes entered on MOXXI electronic health record by the treating physician.

Patients who were not covered by the RAMQ drug insurance plan were excluded, as there would be incomplete information on prior use of OHA. Patients who had a history of gestational diabetes, who were under the age of 18 and who were started on metformin without a diagnosis of diabetes in the past 5 years were excluded from the study.

### Canadian diabetes guideline change

Prior to the new guidelines, overweight patients would be started on metformin as first agent; however, no specific agent was recommended for those who had a BMI of less than 25 [12]. In 2008, metformin became the initial OHA due to its effectiveness in lowering blood glucose without causing weight gain or hypoglycemia [10,11]. In addition, metformin has a good safety profile with minimal side effects.

Each patient in the cohort was classified as being started on therapy in the pre- versus post- 2008 guidelines period, in accordance with the date the drug was prescribed. Although the new CDA guidelines were published in September 2008, we set the date of January 1 2008 as the cut-off for the post-guideline period because guidelines updates are often discussed at conferences prior to the actual publication date (for example, CDA October 24–27, 2007), or by the CDA Guideline Dissemination and Implementation Committee. In addition, evidence of the benefits of metformin (no hypoglycemia or weight gain) was published well before the guidelines publication (UKPDS 34) [11] and information likely disseminated in seminars.

### Patient characteristics

To control for confounding by potential differences in the characteristics of patients started on therapy in the pre and post guidelines change period, we measured age, gender, and renal and cardiovascular co-morbidities (peripheral vascular disease, myocardial infarction, congestive heart failure and cerebrovascular disease) documented in the patient’s electronic medical record.

### Physician characteristics and attitudes to evidence-based practice

To test the hypothesis of whether physicians with evidence-based orientation are more compliant to guidelines, we used the evidence scale from the Evidence-Practicality-Conformity questionnaire completed by each physician at the time of enrollment [19-21]. The evidence scale is created by summing Likert scale ratings from six of the 17 questionnaire items (each question score ranging from 1–5; score range 6–30). Higher scores on this scale have been associated with clinical guidelines compliance and less off-label prescribing in prior studies [19-21]. In addition, we measured physician sex and years of practice experience as both are associated with physicians’ prescribing behaviour [22].

### Outcome

The primary outcome was the choice of initial hypoglycemic therapy, measured using data on prescriptions and dispensed drugs from both the MOXXI and RAMQ database. Oral hypoglycemic agents were grouped into their respective classes: metformin (being the only biguanide was labelled under its generic name), sulfonylurea [(SU) glicalizide, glimeperide, glyburide, tolbutamide], thiazolidinediones [(TZD) pioglitazone, rosiglitazone] and others [alpha-1 glucosidase inhibitor (acarbose), meglitinides (repaglinide), DPP4-inhibitor (sitagliptin)]. We classified each patient as being started on metformin (yes vs. no), as well as by the specific class of therapy they received.

### Analysis

To assess whether there was a difference in the proportion of patients with type 2 diabetes who were initiated on metformin before and after the publication of the 2008 CDA guidelines, we used multivariate logistic regression. As patients were clustered within physician, we used a generalized estimating equation (GEE) framework, where patient was the unit of analysis, physician was the clustering factor, and an exchangeable correlation structure was used to account for dependence among observations. Prescription of metformin (yes vs no) was the binary outcome variable, and patient’s age, sex and the presence or co-morbid cardiovascular and renal disease were included as potential confounders. To assess whether physicians’ with more positive attitudes towards evidence-based practice were more likely to modify their prescribing behaviour in response to a guideline, we included the evidence-based practice score (grouped into tertiles of <20, 20–23, and >23) and guidelines period (pre vs post 2008) as interaction terms in the model. We also included physician gender and practice experience in the model as a potential confounders of evidence-based attitudes.

In secondary analyses, we assessed whether there were differences in the classes of OHA used in the pre and post 2008 guidelines period, with the expectation that any increase in metformin use would be accompanied by a reduction in treatment initiation with other classes of OHA. In this model, treatment period was used as the binary outcome variable (pre vs post 2008 guidelines), and starting class of therapy was used as a multicategorical dummy variable, using metformin as the reference category. The model was adjusted for patient and physician characteristics using multivariate GEE models, as outlined previously. All analyses were performed using SAS software 9.2.

## Results

### Baseline characteristics

Of the 8170 prescribed with an OHA between January 20 2003 and December 29 2011, 5438 adults were fully covered by the RAMQ drug insurance plan, and had no previous diagnosis of gestational diabetes. Of these, 1316 (16.1%) were newly started on an OHA. Within this group, 34 (2.6%) were prescribed metformin without a diagnosis of diabetes. Therefore, 1279 (15.7%) patients were included in the study population. There were a total of 111 physicians in the study. However, a total of 19 physicians did not complete the Evidence-Practicality-Conformity questionnaire, and were excluded from the multivariate analysis of interaction effects.

Overall, 49.2% of the study population were male, 53.4% were over 65 years of age, 7.4% had cardiovascular problems, and 1.3% had renal impairment, 92% were started on one agent (Table 1). Overall, 54.1% of the physicians were male, 58.7% has an evidence score of 20–23. Patients and primary care physicians in the pre versus post guideline periods were similar in their characteristics.

**Table 1:** Characteristics of patients with Type 2 diabetes started on oral hypoglycemic pre and post Canadian Diabetes Association (CDA) 2008 guidelines, and their primary care physicians

	**No (%) of patients or mean ± SD**		
	**Pre**	**Post**	**Overall**
**Patient characteristics**	**n=670**	**n=609**	**n=1,279**
Gender			
• Female	341 (50.9)	309 (50.7)	650 (50.8)
• Male	329 (49.1)	300 (49.3)	629 (49.2)
Age			
• <65	276 (41.2)	282 (46.3)	558 (43.6)
• ≥65	394 (58.8)	327 (53.7)	721 (53.4)
Co-morbidities			
• Cardiovascular	45 (6.7)	40 (6.6)	95 (7.4)
• Renal	10 (1.5)	7 (1.2)	17 (1.3)
**Drugs**			
Number of oral hypoglycemic agents			
• 1	610 (91.0)	567 (93.1)	1177 (92.0)
• ≥2	60 (9.0)	42 (6.9)	102 (8.0)
**Physician characteristics**	**n=95**	**n=73**	**n=111**
Gender			
• Female	45 (47.4)	30 (41.1)	51 (45.9)
• Male	50 (52.6)	43 (58.9)	60 (54.1)
Practice experience (in years) (mean, SD)	22 (8.1)	23.4 (7.6)	21.95 (8.08)
Evidence score	**n=83**	**n=68**	**n=92**
(mean, SD)	21.1 (2.4)	21.2 (2.5)	21.2 (2.51)
• <20	20 (24.7)	18 (26.5)	23 (25.0)
• 20-23	49 (60.5)	38 (55.9)	54 (58.7)
• >23	12 (14.8)	12 (17.7)	15 (16.3)

### Metformin use pre vs post guideline

**Figure 1:**
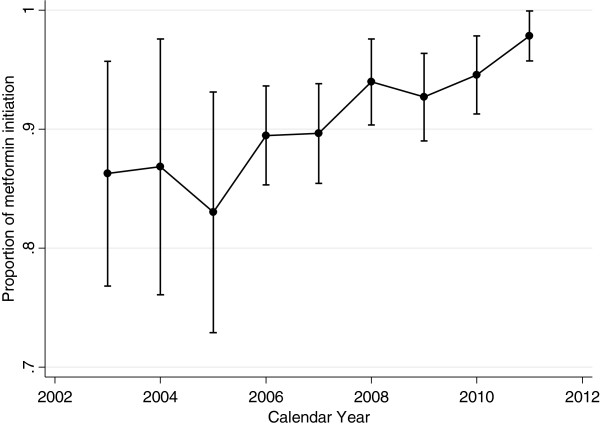
The graph shows the proportions of patients started on metformin (out of the total oral hypoglycemic agents initiated for patients with newly diagnosed type 2 diabetes) and the 95% confidence intervals for calendar years 2003–2011.

After the publication of the new CDA guidelines, there was an absolute increase in incident use of metformin of 4.9% (pre-guidelines 89.7%; post-guidelines 94.6%; OR: 1.86, 95% CI 1.20-2.90) (Table 2 and 3). Metformin use increased even after adjusting for patient and physician characteristics (OR: 1.86, 95% CI 1.20-2.90). Although physicians with higher scores (upper vs. bottom tertile) on the evidence-based practice scale were more likely to prescribe metformin (OR 1.16, 95% CI 0.55-2.46), it was not statistically significant. A trend of increasing use of metformin was seen particularly from 2007 to 2011 compared with 2003 (Figure 1).

**Table 2:** Multivariate analysis of patients’ characteristics and started on metformin (Yes/No)

	**No (%) of patients or mean ± SD**		Multivariate analysis*	
	**Yes**	**No**	**OR (95% CI)**	**p value**
**Patient characteristics**	**n = 1177**	**n = 102**		
Gender				
• Male	582 (49.5)	47 (46.1)	1.00 [Reference]	
• Female	595 (50.6)	55 (53.9)	0.85 (0.55-1.32)	0.48
Age				
• ≥65	649 (55.1)	72 (70.6)	1.0 [Reference]	
• <65	528 (44.9)	30 (29.4)	1.64 (1.09-2.48)	0.02
Co-morbidities				
Cardiovascular				
• No	1101 (93.5)	93 (91.2)	1.00 [Reference]	
• Yes	76 (6.5)	9 (8.8)	0.78 (0.37-1.66)	0.52
Renal				
• No	1167 (99.1)	95 (93.1)	1.00 [Reference]	
• Yes	10 (0.9)	7 (6.9)	0.14 (0.05-0.40)	<0.0001
**Drugs**				
Number of OHA				
• ≥2	93 (7.9)	9 (8.8)	1.00 [Reference]	0.83
• 1	1084 (92.1)	93 (91.2)	0.91 (0.37-2.19)	
**Physician characteristics**	**n=95**	**n=73**		
**MD gender**				
• Male	59 (54.1)	34 (63.0)	1.00 [Reference]	
• Female	50 (45.9)	20 (37.0)	1.21 (0.66-2.23)	0.53
Practice experience (in years) (mean, SD)	21.9 (8.2)	24 (7.8)	1.00 (0.96-1.05)	0.94
MD evidence scale (mean, SD)	**n=90**	**n=52**		
• <20	22 (24.4)	14 (26.9)	1.00 [Reference]	
• 20-23	53 (58.9)	31 (59.6)	0.71 (0.38-1.34)	0.29
• >23	15 (16.7)	7 (13.5)	1.16 (0.55-2.46)	0.69
**Evidence guidelines**^†^				
2008 CDA guidelines				
• Pre	601 (51.1)	69 (67.7)	1.00 [Reference]	
• Post	576 (48.9)	33 (32.4)	1.86 (1.20-2.90)	0.01

Note: *OHA* Oral hypoglycemic agent, *CDA* Canadian Diabetes Association.

*Multivariate analysis done for patients’ gender, age and co-morbidities, guidelines, MD evidence scale, gender, practice experience and drugs.

^†^Model cannot converge when an interaction term involving guideline and MD evidence scale (categorical) were included.

**Table 3:** Multivariate analysis for OHA classes pre and post CDA 2008 guidelines

	**No (%) of patients or mean ± SD**		Multivariate analysis*	
	**Pre**	**Post**	**OR (95% CI)**	**p value**
	**n = 670**	**n = 609**		
Classes of OHA^†^				
• Metformin ± other^‡^	601 (89.7)	576 (94.6)	1.00 [Reference]	
• TZD	18 (2.7)	3 (0.5)	0.21 (0.08-0.55)	0.001
• Sulfonylurea	54 (8.1)	27 (4.4)	0.68 (0.43-1.09)	0.11
• Other^‡^	3 (0.5)	4 (0.7)	1.44 (0.29-7.06)	0.65

Note: *OHA* Oral hypoglycemic agent, *TZD* Thiazolidinediones, *CDA* Canadian Diabetes Association.

*Multivariate analysis done for patients’ gender, age and co-morbidities, guidelines, MD evidence scale, gender, practice experience and drugs.

^†^Patients initiated on more than 1 drug (with no Metformin) were counted in both classes.

^‡^Includes Alpha-1 glucosidase inhibitor (Acarbose), DPP-4 inhibitor (Sitagliptin) and Meglitinides.

Among the 1177 (92.0%) patients who were started on metformin, patients younger than 65 years old had 64% more odds in receiving metformin (OR 1.64, 95% CI 1.09-2.48). In addition, metformin was less likely to be used among patients with renal disease (OR 0.14; 95% CI 0.05-0.40). Concurrent with an increase in the use of metformin post-guidelines, there was a 79% reduction in the odds of initiation of TZD (OR 0.21, 95% CI 0.08-0.55) and a statistically non-significant 32% decrease in the odds of treatment initiation with SU (OR 0.68, 95% CI 0.43-1.09) (Table 3).

## Discussion

To our knowledge, this is the first study to examine how a change in guidelines for diabetes treatment was temporally associated with the choice of starting therapy for newly treated type 2 diabetics. Pre-2008 guidelines, similar to the population-based Ontario study [13], our paper showed a high initiation rate of metformin. Post-guidelines, we showed an absolute increase of 4.9% in its use. This higher initiation of metformin was associated with a decreased prescription of thiazolidinediones and sulfonylureas, which suggested physicians’ compliance to the change of guidelines. However, around the same period, in 2007–2008, some studies showed an association of rosiglitazone with an increased risk of myocardial infarction and a possible increase in the risk of death from cardiovascular causes [23,24]. Subsequently, a black box warning issued in October 2007 by drug regulatory agencies in both Canada and the United States may have been an important contributing cause to change in prescribing practices. However, given both TZD and SU decreased (although not statistically significant due to the small sample size), accompanied by a rise in metformin initiation, it is plausible that a change in treatment guidelines contributed to this trend. It addition, it was also seen by Shah et al. [25] that the major decline of TZD actually occurred in 2007.

Guidelines are published every few years with the aim to help physicians incorporate new scientific evidence into clinical practice and improve the standard of care. However, the translation of evidence into clinical practice is often slow, unpredictable and incomplete [26]. Prior studies have demonstrated only mild to moderate improvement in patients’ care post-guideline dissemination [27-29]. In the case of diabetes, some studies investigating physicians’ adherence to diabetes guidelines revealed low to moderate compliance [8,30-33]. This is likely due to the complexity of the disease and by the number of medications required [9]. Contrary to these studies, our study demonstrated high initiation of metformin in incident patients with type 2 diabetes, and a further increase post-guidelines change. In addition, when yearly metformin initiation rate was analyzed, we saw an increasing trend since 2007. This higher compliance rate could be due to the abundance of research published prior to the guidelines indicating the benefits of metformin [11,34].

As expected, the initiation of metformin was lower in those with renal failure and who are older than 65 years old. The use of metformin in the elderly has been heavily debated [35,36]. The lower use could be due to the reluctance of physicians to prescribe to the elderly due to the side effects related to metformin (gastrointestinal upset, including diarrhea, nausea, vomiting and flatulence) and the rare possibility of lactic acidosis in those with renal failure [37]. While age created a difference in drug treatment choice, more evidence-based physicians were not more likely to change practice. This could be due to the high initiation rate of metformin (89.7%) pre-guideline, as well as limited power to detect differences in effect.

### Strengths and limitations

To our knowledge, our study is the first to examine how a change in guidelines for diabetes treatment was temporally associated with the choice of starting therapy for newly treated type 2 diabetics. We were able to include a large number of patients over eight years. Having the ability to examine physicians’ prescriptions instead of administrative data that represent drugs dispensed enabled us to look at the physicians’ actual prescribing decisions and exclude medication compliance bias.

Our study has several limitations. This research used a selective cohort of primary physicians: only primary care physicians who elected to use an electronic medical record system, and their knowledge and intended use of the new CDA guidelines was not measured. Also, patients who were not fully covered by RAMQ drug insurance were excluded (33.2% of the adult diabetic population); thus, this could exclude patients who were younger (in Quebec, patients older than 65 are mostly covered by RAMQ [14]) or who had higher socio-economic status, where prescribing decisions could have been different. In addition, the use of metformin in patients with incident diabetes was already high prior to guidelines, making the impact less than predicted despite the change in guideline. An analysis of metformin prescription rates for obese versus non-obese individuals would have been valuable, but could not be performed due to the lack of patients’ body mass index in the MOXXI database.

Our study showed that there was only a small percentage of patients with co-morbid illness (7.4% patients with cardiovascular disease and 1.3% with renal illness). This could either be attributed to healthier primary care population than those typically studied in hospital populations, or alternatively, incomplete chart documentation of co-morbid conditions.

## Conclusions

Unlike previous studies showing low adherence to guidelines [8,38], this study demonstrated an increase in the initiation of metformin as first line treatment instead of sulfonylurea or thiazolidinediones by primary care physicians after the new CDA 2008 guidelines. However, due to the nature of the study design, we can not determine whether the observed change in metformin prescribing was causally related to the change in the guideline. The next step would be to examine the compliance in other aspect of diabetes management and sustainability of guidelines adherence among primary care physicians and specialists.

## Competing interests

There are no author conflicts to declare with regard to this manuscript.

## Authors’ contributions

TW, TE, and RT had full access to all of the data in the study and take responsibility for the integrity of the data and the accuracy of the data analysis. TW developed the study concept and design, wrote the manuscript and researched data. TE developed the study concept and design, reviewed/edited the manuscript. TE is supported by the Canadian Institutes of Health Research (CIHR) Postdoctoral fellowship and CIHR Emerging Team Grant. RT developed the study concept and design, reviewed/edited the manuscript, supervised the study and obtained funding (Funding Sources: CIHR). All authors read and approved the final manuscript.

## Pre-publication history

The pre-publication history for this paper can be accessed here:

http://www.biomedcentral.com/1472-6963/13/442/prepub
